# Dexmedetomidine Attenuates Oxidative Stress Induced Lung Alveolar Epithelial Cell Apoptosis *In Vitro*

**DOI:** 10.1155/2015/358396

**Published:** 2015-03-08

**Authors:** Jian Cui, Hailin Zhao, Chunyan Wang, James J. Sun, Kaizhi Lu, Daqing Ma

**Affiliations:** ^1^Department of Anaesthesiology, Southwest Hospital, Third Military Medical University, Chongqing 400038, China; ^2^Section of Anaesthetics, Pain Medicine and Intensive Care, Department of Surgery and Cancer, Faculty of Medicine, Imperial College London, Chelsea and Westminster Hospital, London SW10 9NH, UK

## Abstract

*Background*. Oxidative stress plays a pivotal role in the lung injuries of critical ill patients. This study investigates the protection conferred by *α*_2_ adrenoceptor agonist dexmedetomidine (Dex) from lung alveolar epithelial cell injury induced by hydrogen peroxide (H_2_O_2_) and the underlying mechanisms. *Methods*. The lung alveolar epithelial cell line, A549, was cultured and then treated with 500 *μ*M H_2_O_2_ with or without Dex (1 nM) or Dex in combination with atipamezole (10 nM), an antagonist of *α*_2_ receptors. Their effect on mitochondrial membrane potential (Δ*ψ*_m_), reactive oxygen species (ROS), and the cell cycle was assessed by flow cytometry. Cleaved-caspases 3 and 9, BAX, Bcl-2, phospho-mTOR (p-mTOR), ERK1/2, and E-cadherin expression were also determined with immunocytochemistry. *Results*. Upregulation of cleaved-caspases 3 and 9 and BAX and downregulation of Bcl-2, p-mTOR, and E-cadherin were found following H_2_O_2_ treatment, and all of these were reversed by Dex. Dex also prevented the ROS generation, cytochrome C release, and cell cycle arrest induced by H_2_O_2_. The effects of Dex were partially reversed by atipamezole. *Conclusion*. Our study demonstrated that Dex protected lung alveolar epithelial cells from apoptotic injury, cell cycle arrest, and loss of cell adhesion induced by H_2_O_2_ through enhancing the cell survival and proliferation.

## 1. Introduction

Oxidative stress plays a pivotal role in acute lung injury (ALI) and acute respiratory distress syndrome (ARDS), which is common in critically ill patients undergoing mechanical ventilation [1, 2]. The source of oxidative stress for lung cells may be intrinsic or extrinsic. Oxidants generated intrinsically are from the mitochondria of injured alveolar epithelial cells. Extrinsic oxidants may arise from air inhalation or generated from phagocytic cells [3, 4]. Phagocytic cells can generate free radicals which act on the cell surfaces to form nicotinamide adenine dinucleotide phosphate (NADP^+^), an oxidant, and this process often occurs in vascular endothelial cells and lung alveolar epithelial cells [5].

The degradation of the oxidizing products depends on the antioxidant enzymes including superoxide dismutase (SOD), catalase, glutathione peroxidase, and glutathione [6]. SOD catalyses the dismutation of the superoxide anion to hydrogen peroxide (H_2_O_2_). Catalase converts H_2_O_2_ to H_2_O in the presence of glutathione, which is then converted to its oxidized form, glutathione disulfide (GSSG). The presence of these antioxidant enzymes is important in maintaining a redox balance in our body and so that one can respond to oxidizing conditions which may threaten the structural and functional integrity of the vital organs including the lungs [7]. In a variety of situations such as severe liver disease, cachexia, and alimentary deficiency, the antioxidant enzymes are insufficient to clear the oxidation products [8]. Lung alveolar epithelial cells, especially type II alveolar cells, are particularly sensitive to oxidative stress [9]. Epithelial cell apoptosis is followed by remodeling processes, which consist of epithelial and fibroblast activation, cytokine production, activation of the coagulation pathway, neoangiogenesis, reepithelialization, and fibrosis [10]; therefore, lung injury is often present at the end stage of liver failure [11].

Recent studies have demonstrated that dexmedetomidine (Dex), a potent *α*_2_ adrenergic agonist with sedative, analgesic, sympatholytic, and hemodynamic effects, can reduce systemic inflammation and improve diaphragmatic function and gas exchange in perioperative patients [12, 13]. We hypothesized that protective signaling pathways in lung cells might be activated by Dex to prevent cellular apoptosis when subjected to oxidative insult. In this study, we aim to investigate whether Dex protects the lung alveolar epithelial cells against H_2_O_2_-induced oxidative stress.

## 2. Material and Methods

### 2.1. Cell Culture and Drug Treatments

The A549 human alveolar epithelial cells (European Cell Culture Collection, UK) were cultured in RPMI 1640 (Abcam, Cambridge, UK) medium supplemented with 10% fetal bovine serum (FBS, Abcam) at 37°C in 5% CO_2_. Cells were challenged with 500 *μ*M H_2_O_2_ (Sigma-Aldrich, Dorset, UK) in fresh RPMI 1640 medium without FBS for 24 hours in the control group, while, in the treatment groups, the cells were pretreated with 1 nM Dex (Orion Pharm Ltd., Newbury, Berkshire, UK) for 15 minutes, in the presence or absence of 10 nM atipamezole (Sigma Aldrich, Cambridge, UK), a synthetic *α*_2_-adrenergic antagonist and then exposed to 500 *μ*M H_2_O_2_ for additional 24 hours.

### 2.2. Immunocytochemistry

The naive or treated A549 cells were blocked with donkey serum and then incubated with the following primary antibodies: rabbit anti-human BAX, Bcl-2, cleaved-caspase 9, caspase 3, p-mTOR, ERK1/2, and E-cadherin (1 : 250, Abcam) overnight followed by fluorochrome conjugated donkey anti-rabbit secondary antibody for 1 h. The nuclei were counterstained with 4′,6-diamidino-2-phenylindole (DAPI, Invitrogen, Warrington, UK) and examined with an Olympus BX40 microphotography system (Rochester NY, USA). The mean intensity of fluorescence obtained from ten samples was analyzed with Image J software (NIH, US National Institutes of Health, Bethesda, MD).

### 2.3. Determination of Δ*ψ*_m_

The cells were labeled with 5,5′,6,6′-tetrachloro-1,1achlortetrathylbenzimidazolyl-carbocyanine iodide (JC-1) dye (e-bioscience, Ireland, UK) and assessed by flow cytometry and microscope, respectively [14]. For FACS detection, cells were detached using 0.25% trypsin and then transferred to 5 mL polystyrene tubes. After washing with FACS buffer once, the cells were incubated with 0.2 *μ*M JC-1 in FACS for 30 minutes at 37°C and protected from light. The cells were analyzed by flow cytometry after being washed with warm FACS buffer twice. Fluorescence was measured in the FL1 (FITC) and FL2 (PE) channels, gating only living cells. The mean intensity of red fluorescence (PE) and green fluorescence (FITC) was analyzed using FlowJo 7.6.1 software (TreeStar, San Carlos, CA) and then the ratio of red/green fluorescence intensity was analyzed. A decrease in the red/green fluorescence ratio is indicative of mitochondrial depolarization. To further investigate the effect of Dex and H_2_O_2_ on the changes of cell Δ*ψ*_m_, cells stained with JC-1 dye were also visualized by microscope. In brief, cells were incubated with 10 *μ*M JC-1 dye in FACS buffer for 30 minutes at 37°C after being washed with warm 0.1 M PBS once. Then, the nuclei were stained using DAPI. The fluorescence staining was examined under rhodamine (red), fluorescein (green), and cyan (blue) spectral filters with the Olympus BX40 microphotography system.

### 2.4. Determination of ROS Production by Flow Cytometry

ROS production was determined by 2′-7′-dichlorodihydrofluorescein diacetate (DCF) staining and the intensity of fluorescence was assessed by flow cytometry [15]. A549 cells were harvested by trypsinization and washed with FACS buffer. The cells were incubated in 2 *μ*M DCF diluted in FACS buffer for 30 minutes at 37°C. The fluorescence was assessed by flow cytometry (FACS Calibur; Becton Dickinson, Sunnyvale, CA) and analyzed with FlowJo 7.6.1 software. Each assay included at least 10,000 gated events.

### 2.5. Cell Cycle Analysis by Flow Cytometry

The cell cycle was analyzed by flow cytometry as described previously [16]. The cells were fixed with 70% ethanol at 4°C overnight. After centrifugation at 2500 rpm for 10 minutes and resuspension in 500 *μ*L PBS, 10 *μ*L of 500 ng/L RNase and 10 *μ*L of 40 *μ*g/L PI were added to the cell suspension and then incubated for 10 minutes at room temperature. The fluorescence of PI uptake in cells was detected with flow cytometry and analyzed with FlowJo 7.6.1 software. Each assay included at least 10,000 gated events.

All of the above experiments have been repeated more than 8 times independently.

## 3. Statistical Analysis

All numerical data is presented as mean ± SD. Comparison between the treatment groups was analyzed by one-way ANOVA of variance, followed by Tukey's test (GraphPad prism 5, San Diego, CA, USA). A *P* value of 0.05 was considered as statistically significant.

## 4. Results

### 4.1. Effect of Dex on the Expression of Cleaved-Caspases 3 and 9, BAX, and Bcl-2 following H_2_O_2_ Challenge

**Figure 1:**
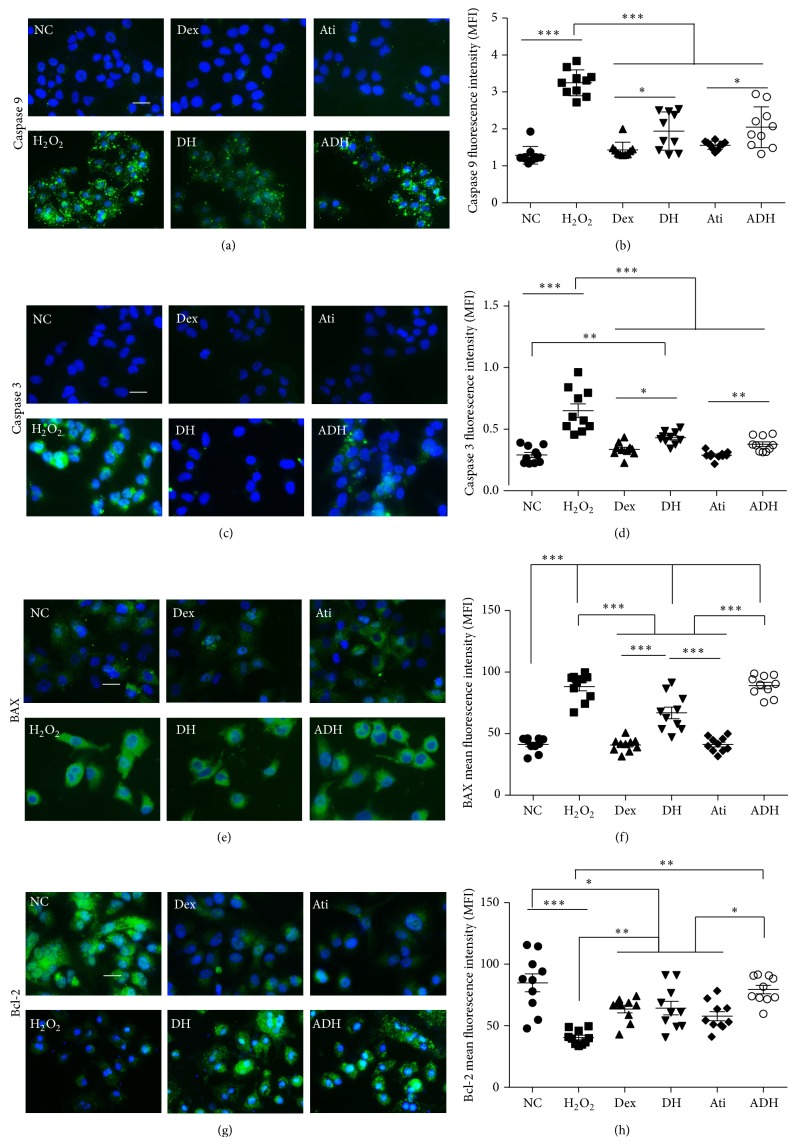
Effect of Dex on the expression of cleaved-caspases 3, 9, BAX, and Bcl-2 in A549 cells. A549 cells were treated with 500 *μ*M H_2_O_2_ for 24 hours. Cohort cultures received 1 nM Dex together with or without 10 nM atipamezole for 15 minutes and then treated with H_2_O_2_ for additional 24 hours. (a) Expression of cleaved-caspase 9 (green); (b) fluorescence intensity of cleaved-caspase 9; (c) expression of cleaved-caspase 3 (green); (d) fluorescence intensity of cleaved-caspase 3; (e) expression of BAX in A549 cells assessed by immunofluorescence staining (green); (f) mean fluorescence intensity of BAX; (g) expression of Bcl-2 in A549 cells assessed by immunofluorescent staining (green); (h) mean fluorescence intensity of Bcl-2. Scale bar = 50 *μ*m. ^*^ *P* < 0.05; ^**^ *P* < 0.01; ^***^ *P* < 0.001; *n* = 10. NC: naïve control; Dex: dexmedetomidine; Ati: atipamezole; DH: A549 cells treated with H_2_O_2_ followed by Dex; ADH: A549 cells treated with H_2_O_2_ following by Dex and Ati.

Expression of apoptotic proteins cleaved-caspase 3, caspase 9, and the proapoptotic protein BAX were upregulated in the A549 cells exposed to H_2_O_2_. The expression of antiapoptotic protein Bcl-2 in A549 cells was downregulated when detected with immunocytochemistry following the challenge of 500 *μ*M H_2_O_2_  for 24 hours, and all these effects were partially reversed by the pretreatment with 1 nM Dex. Atipamezole at a dose of 10 nM only partially reversed the effects of Dex on the expression of BAX and Bcl-2 and had no effects on that of cleaved-caspases 3 and 9 (Figure 1).

### 4.2. Effect of Dex on the Δ*ψ*_m_ Collapse and ROS Generation and Cytochrome C Release Induced by H_2_O_2_

**Figure 2:**
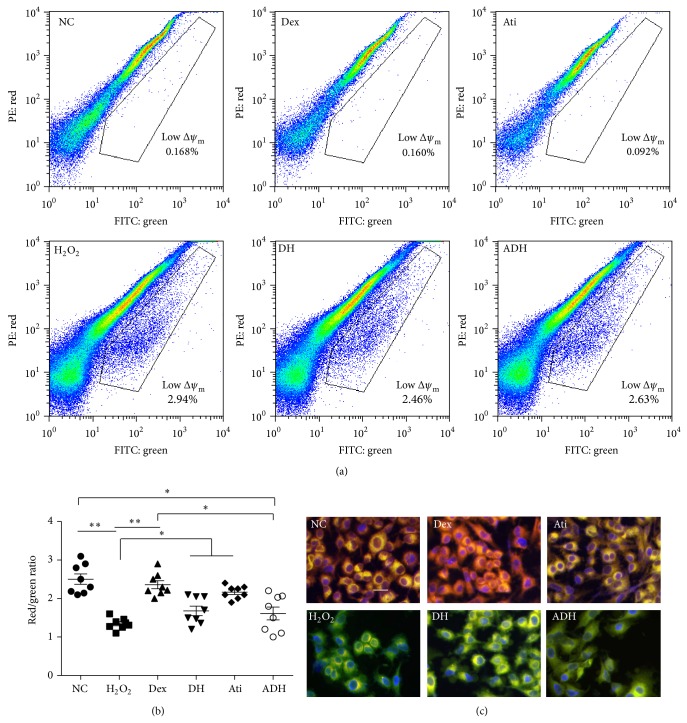
Effects of Dex on H_2_O_2_-induced Δ*ψ*_m_ collapse in A549 cells. (a) Δ*ψ*_m_ was measured by flow cytometry under FL1 (FITC) and FL2 (PE) channel after being stained with JC-1. The cells in gating areas with low ratio of red/green fluorescence intensity indicated cells with low Δ*ψ*_m_; (b) ratio of fluorescence intensity of JC-1 (red/green), assessed by flow cytometry. ^*^ *P* < 0.05, ^**^ *P* < 0.01; *n* = 8; (c) A549 cells were staining with JC-1 and nuclei were counterstained with DAPI. The cells were examined with microscope under rhodamine (red), fluorescein (green), and cyan (blue) spectral filters. The figures are merged from the three colors. Mitochondrial depolarization was indicated by a decreased red fluorescence and increased green fluorescence. Scale bar = 50 *μ*m. PE: phycoerythrin; FITC: fluorescein isothiocyanate.

**Figure 3:**
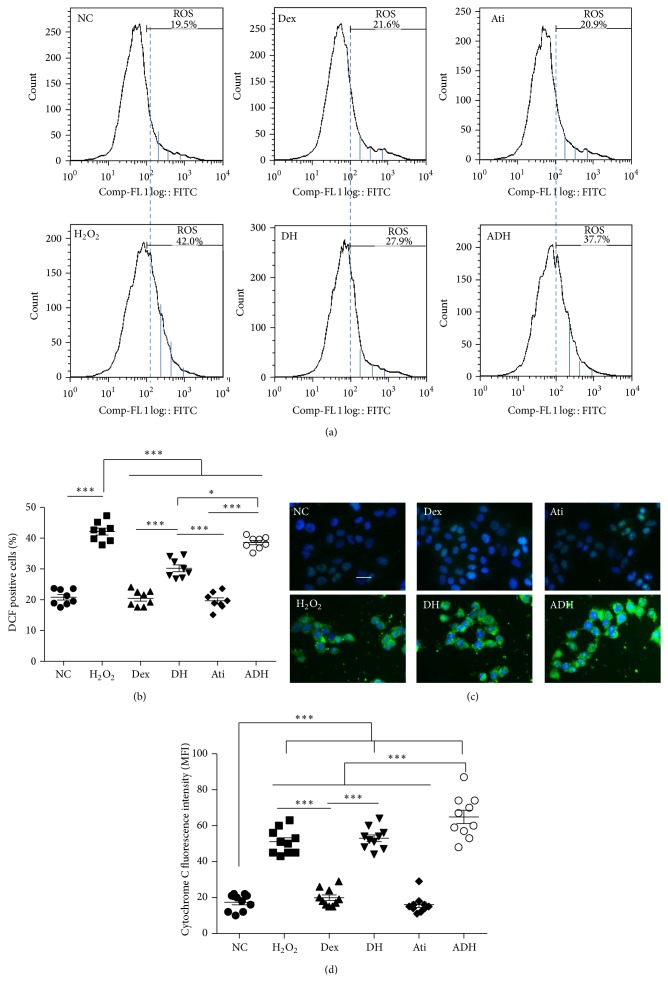
Dex reversed the ROS generation and cytochrome C release in A549 cells induced by H_2_O_2_. (a) ROS production detected by flow cytometry after DCF staining. The increase of the FITC fluorescence intensity indicated the increase in percentage of DCF positive cells; (b) percentage of the DCF positive cells, *n* = 8; (c) Overexpression of cytochrome C in A549 cells was not reversed by Dex and was enhanced by atipamezole, Dex treated together; (d) mean fluorescence intensity of cytochrome C. Scale bar = 50 *μ*m. ^***^ *P* < 0.001; *n* = 10. DCF: 2′-7′-dichlorodihydrofluorescein diacetate.

Compared with that of naïve control cells, the Δ*ψ*_m_ of mitochondria in H_2_O_2_ treated A549 cells decreased significantly, and this effect was also abolished by Dex when detected by flow cytometry and microscopy. There was no significant difference in Δ*ψ*_m_ between Dex and Dex combined with atipamezole pretreated cells (Figure 2). The percentage of cells with high ROS production increased significantly after being challenged with 500 *μ*M H_2_O_2_ for 24 hours when detected with flow cytometry. This was reversed by pretreatment with 1 nM Dex (percentage of DCF positive cells decreased from 43.08 ± 3.15% in H_2_O_2_ treated cells to 30.28 ± 3.49% in H_2_O_2_ combined Dex treated cells, *P* < 0.001). Atipamezole attenuated the effect of Dex on the prevention of ROS generation induced by H_2_O_2_ (Figures 3(a) and 3(b)). H_2_O_2_-induced an increase in the release of cytochrome C from the mitochondria in A549 cells, but there was no significant difference between H_2_O_2_ treated cells and H_2_O_2_ combined Dex treated cells (51.11 ± 6.89 in H_2_O_2_ treated cells versus 53.21 ± 6.04 in H_2_O_2_ combined Dex treated cells, *P* < 0.05). Pretreatment with atipamezole enhanced the release of cytochrome C compared to H_2_O_2_ and Dex combined H_2_O_2_ treated cells (Figures 3(c) and 3(d)).

### 4.3. Effect of Dex on the Activation of mTOR/ERK1/2 Pathway and Cell Cycle Arrest Induced by H_2_O_2_ in A549

**Figure 4:**
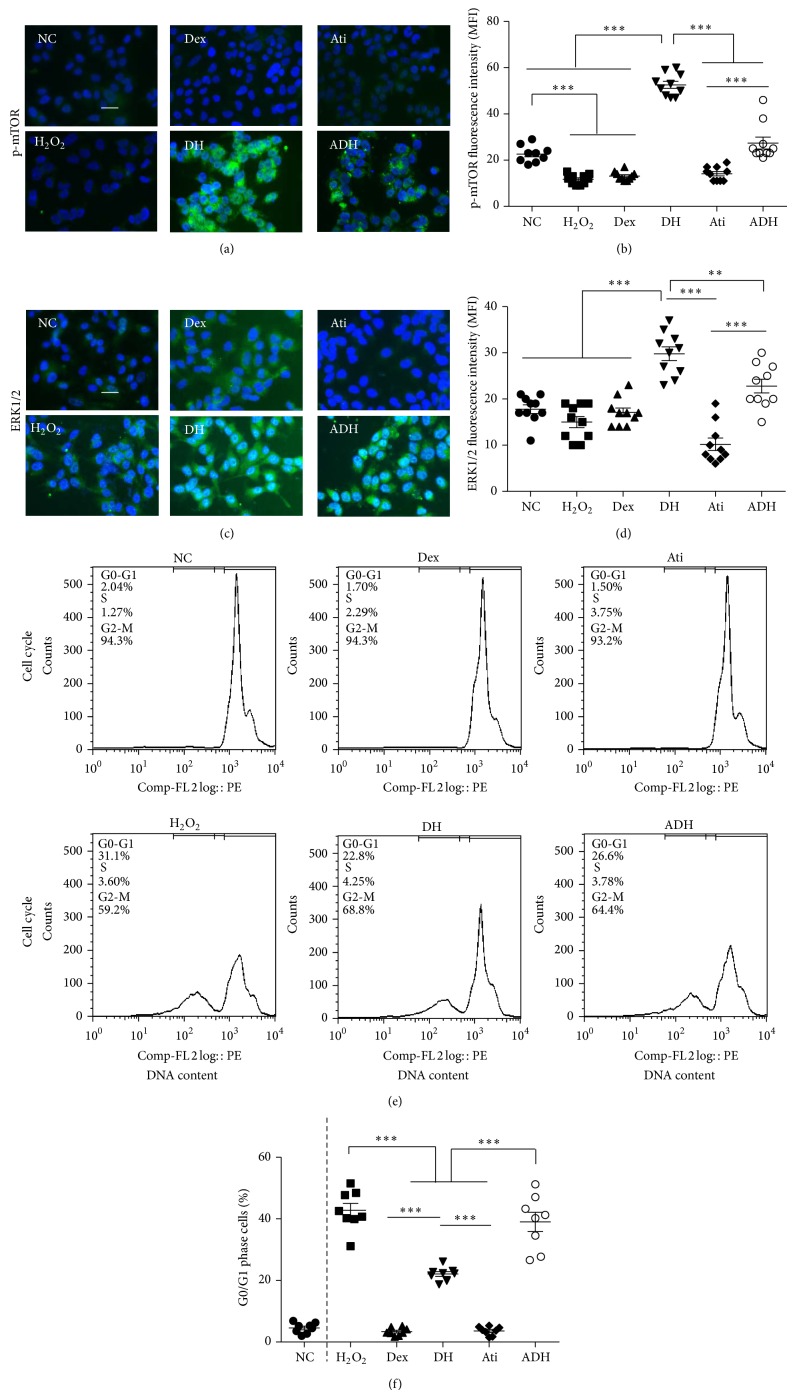
Dex prevented the downregulation of p-mTOR, ERK1/2, and cell cycle arrest induced by H_2_O_2_, which was reversed by atipamezole. (a) Expression of p-mTOR in A549 cells assessed by immunofluorescent staining (green); (b) mean fluorescence intensity of p-mTOR; (c) expression of ERK1/2 in A549 cells assessed by immunofluorescent staining (green); (d) mean fluorescence intensity of ERK1/2. Scale bar = 50 *μ*m; *n* = 10; (e) cell cycle analyzed by flow cytometry. Dex prevented the G0/G1 arrest of A549 cells induced by H_2_O_2_ and attenuated by atipamezole; (f) scatter plots of mean percentage of G0/G1 cells; *n* = 8. ^**^ *P* < 0.01, ^***^ *P* < 0.001.

The activation of mTOR and ERK1/2 mediated cell proliferation/survival pathway was investigated by immunostaining to explore the effect of Dex on H_2_O_2_-induced cell proliferation and survival. Expression of p-mTOR (activated mTOR) was reduced and there was no change in ERK1/2 levels after the H_2_O_2_ challenge. Pretreatment with Dex enhanced the expression of p-mTOR and ERK1/2 compared to that in H_2_O_2_ challenged cells, which was partially reversed by atipamezole (fluorescence intensity of p-mTOR: 52.60 ± 4.84 in the DH group compared to 11.70 ± 2.11 in H_2_O_2_ group and 27.41 ± 8.09 in the ADH group, *P* < 0.001; ERK1/2: 29.80 ± 4.69 in the DH group compared to 15.00 ± 3.74 in H_2_O_2_ group and 22.83 ± 4.73 in the ADH group, *P* < 0.01) (Figures 4(a) and 4(b)).

Flow cytometry was used to investigate the effect of Dex on cell cycle progression following H_2_O_2_ insult. Compared with naïve control cells, a greater percentage of cells were arrested at G0/G1 following the H_2_O_2_ challenge (4.43 ± 2.08% in naïve control cells versus 43.40 ± 8.99% in H_2_O_2_ treated cells, *P* < 0.001). Dex attenuated the cell cycle arrest at G0/G1 phase induced by H_2_O_2_ and this effect was partially abolished by 10 nM atipamezole (22.60 ± 6.62% in DH group versus 39.83 ± 10.11% in ADH group, *P* < 0.001) (Figures 4(e) and 4(f)).

### 4.4. Effect of Dex on the Expression of E-Cadherin in A549 Cells after H_2_O_2_ Insult

**Figure 5:**
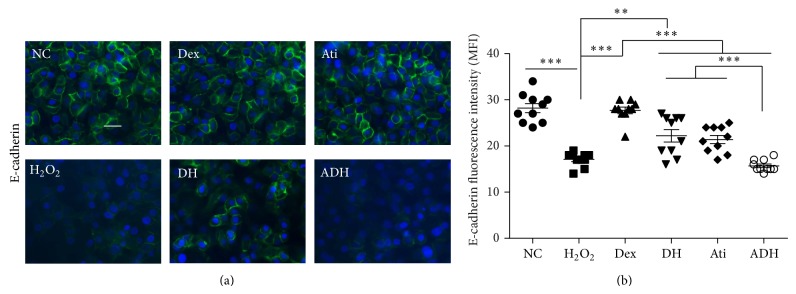
Effect of Dex on the expression of E-cadherin in A549 cells following the challenge of H_2_O_2_. (a) Expression of E-cadherin in A549 cells assessed by immunofluorescent staining (green); (b) fluorescence intensity of E-cadherin. Scale bar = 50 *μ*m. ^*^ *P* < 0.01, ^**^ *P* < 0.01, and ^***^ *P* < 0.001. *n* = 10.

Expression of E-cadherin, which maintains cell adhesion and forms cell-cell junctions to bind cells together, was found to be significantly reduced following the H_2_O_2_ challenge; this was partially reversed by pretreatment with Dex (22.27 ± 4.24 in DH group compared to 17.12 ± 1.52 in H_2_O_2_ group, *P* < 0.01). Atipamezole reversed the inhibition of Dex on the downregulation of E-cadherin induced by H_2_O_2_ (15.77 ± 1.25 in ADH versus 22.27 ± 4.24 in the DH group, *P* < 0.001) (Figure 5).

## 5. Discussion

**Figure 6:**
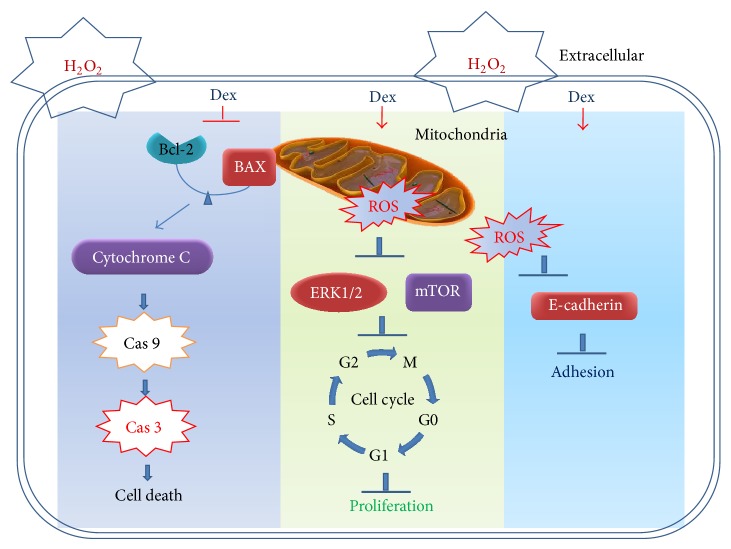
Possible molecular mechanism of Dex-mediated protection against oxidative stress in A549 cell death. H_2_O_2_-induced A549 cell death, cell cycle arrest, and loss of cellular adhesion, which was attenuated by Dex* via* inhibiting the ROS production. ↓ enhanced; ⊥ attenuated. Cas 3: caspase 3; Cas 9: caspase 9.

The present study, for the first time, explores the effect of Dex, a potent *α*_2_ adrenergic agonist, on H_2_O_2_-induced lung alveolar epithelial cell injury. Our results demonstrated that (1) cell injury and cell cycle arrest of A549 cells was found after the 24-hour H_2_O_2_ challenge; (2) Dex attenuated the cell injury and enhanced cell survival following the H_2_O_2_ insult; (3) the protective effect of Dex on H_2_O_2_-induced cell injury was partially dependent on *α*_2_ adrenoceptor activation (Figure 6).

One important aspect of ALI is the oxidative stress to the lungs mediated by ROS [4]. Biologically significant ROS include superoxide anion radical (O_2_^−^), H_2_O_2_, hydroxyl radical (OH^−^), and hypohalous acids such as hypochlorous acid (HOCI) [17]. In this study, we used H_2_O_2_ to challenge the lung alveolar epithelial A549 cells and explored the relationship between oxidative stress and epithelial cell injury. Following the challenge of H_2_O_2_ on A549 cells for 24 hours, proapoptotic proteins such as cleaved-caspases 3 and 9 and BAX were upregulated and antiapoptotic protein Bcl-2 was downregulated; all of these results indicated that 500 *μ*M H_2_O_2_ was sufficient to activate the cell intrinsic apoptotic pathway (Figure 1). Upstream of the caspase mediated apoptotic pathway and mitochondrial membrane potential (Δ*ψ*_m_) decreased and ROS generation accompanied by cytochrome C release increased following H_2_O_2_ challenge (Figures 2 and 3). Mitochondrial dysfunction and toxic substance accumulation in the cytoplasm activated caspase mediated programmed cell death [18]. The inhibitory effects of Dex on the upregulation of apoptotic proteins, downregulation of antiapoptotic proteins, and reduction of Δ*ψ*_m_ identified its protective effects on oxidative stress induced cell death which at least in part is due to the activation of the *α*_2_ adrenoceptor (Figure 2).

Cell cycle arrest in alveolar epithelial cells induced by H_2_O_2_ indicated that cell proliferation and further repair of the damaged epithelium were inhibited [19]. The mTOR/ERK1/2 proliferation/survival pathway is activated, which was enhanced by pretreatment with Dex in A549 cells following H_2_O_2_ challenge. This indicated that Dex may exert its potent effect on promoting the repair of the injured epithelium following oxidative stress (Figure 4). Cell surface adhesion molecule E-cadherin is linked internally with cytoskeletal components and provides linkage between the cell membranes and thus is essential for cell-to-cell attachment [20]. Dex enhanced the expression of E-cadherin during H_2_O_2_ challenge, indicating that Dex also protects the alveolar epithelial cells from damage at the cell junctions; this may be beneficial in maintaining the integrity of epithelial barrier [21] (Figure 5).

Dex is a potent *α*_2_ adrenergic agonist and it also binds to imidazoline receptors recognizing the imidazoline or oxazoline structure of *α*_2_ agents [22]. *α*_2_ receptors have also been classified into three subtypes (A, B, and C) according to radioligand binding studies and their pharmacokinetic profiles [23]. The molecular mechanism of *α*_2_ receptors is still not clear and it may be due to the activation of inhibitory G proteins, membrane-bound ion channels, and the nitric oxide-cGMP pathway [22]. In this study, we used a potent nonspecific antagonist of *α*_2_ receptors, atipamezole, to block the activation of *α*_2_ receptors, but it did not completely reverse the inhibitory effect of Dex on H_2_O_2_-induced A549 cell apoptosis and cell cycle arrest. These results indicated that imidazoline receptors may be involved in antiapoptotic effect of Dex, but the dose response relationship remains unknown. More studies are needed to determine whether atipamezole binds to imidazoline receptors.

We have clearly demonstrated the effects of Dex on oxidative stress induced lung cell injury and death in our study. However, there are some limitations which warrant further study: (1) using specific inhibitors to inhibit the signaling pathways could better explore the role of such pathways in the cell's fate; (2) other antioxidants could be used to compare its effects with those elicited by Dex; (3) Dex-mediated protective effect via its antioxidative effect is likely one of the mechanisms responsible for oxidative stress induced cell apoptosis. Further studies are required to characterize the mechanism, including its effect on antioxidant enzymes (SOD, GPX, and CAT). This protective effect must be validated in a large animal model and clinical trials.

Dex is a sedative used by intensive care units and anaesthetists to reduce the morbidity of patients under mechanical ventilation [24]. Some clinical trials have that indicated Dex is beneficial for patients due to the reduction in mechanical ventilation time [25–27]. Although not clearly understood, it is possible that less time weaning off the ventilator is associated with a reduced level of lung injuries [28]. However, caution must be taken. (1) This is an* in vitro* study, and further proper* in vivo* study is required. (2) The injurious insult is a “single” one rather than multiple challenges. Nevertheless, our data indicated that Dex represents a promising anaesthetic/sedative choice in protecting patients from ALI under oxidative stress insult, though this warrants further study.

In summary, this study demonstrated that Dex attenuated the H_2_O_2_-induced lung alveolar epithelial cell injury* in vitro*. Although further studies particularly* in vivo* studies followed by clinical trials are needed to further validate the protective effects of Dex on lung injury, its inhibitory effect on cell apoptosis and promotion of cell survival represent a promising anaesthetic/sedative choice in treating the patients with lung injury.
