# Correction to “Dexmedetomidine Attenuates Oxidative Stress Induced Lung Alveolar Epithelial Cell Apoptosis *In Vitro*”

**DOI:** 10.1155/omcl/9890530

**Published:** 2026-01-07

J. Cui, H. Zhao, C. Wang, J. J. Sun, K. Lu, and D. Ma, “Dexmedetomidine Attenuates Oxidative Stress Induced Lung Alveolar Epithelial Cell Apoptosis *In Vitro*,” *Oxidative Medicine and Cellular Longevity* (2015): 358396, https://doi.org/10.1155/2015/358396.

In the article, there is an error in Figure 5a, where there are overlapping regions between the NC and Dex panels. The correct Figure 5 is shown below:

Figure 5Effect of Dex on the expression of E‐cadherin in A549 cells following the challenge of H_2_O_2_. (a) Expression of E‐cadherin in A549 cells assessed by immunofluorescent staining (green); (b) fluorescence intensity of E‐cadherin. Scale bar = 50 μm.  ^∗^ *P* < 0.01,  ^∗^^∗^ *P* < 0.01, and  ^∗^^∗^^∗^ *P* < 0.001. *n* = 10.

**Figure:**
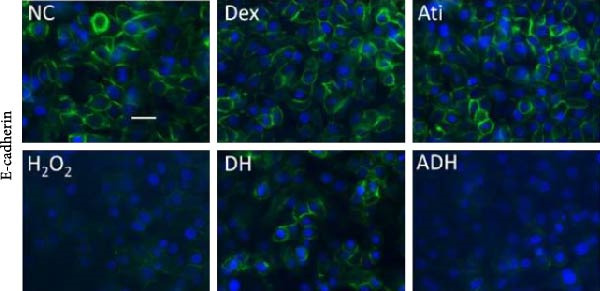
(a)

**Figure:**
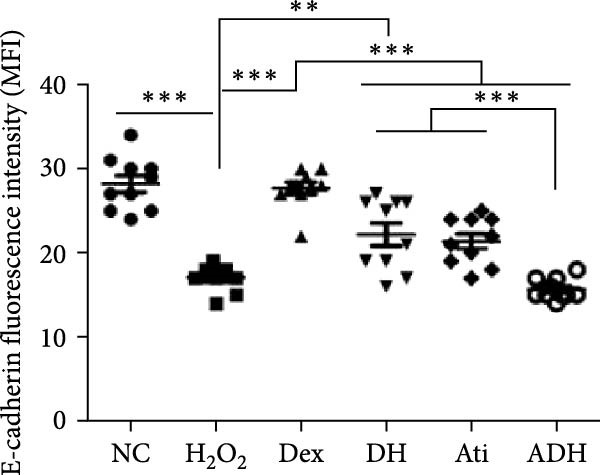
(b)

We apologize for this error.

